# The Association between Early-Life Gut Microbiota and Long-Term Health and Diseases

**DOI:** 10.3390/jcm10030459

**Published:** 2021-01-25

**Authors:** Anujit Sarkar, Ji Youn Yoo, Samia Valeria Ozorio Dutra, Katherine H. Morgan, Maureen Groer

**Affiliations:** 1 College of Nursing, University of South Florida, Tampa, FL 33612, USA; anujit@usf.edu (A.S.); jiyounyoo@usf.edu (J.Y.Y.); 2 College of Public Health, University of South Florida, Tampa, FL 33612, USA; 3 College of Nursing, University of Tennessee- Knoxville, Knoxville, TN 37916, USA; sozoriod@utk.edu (S.V.O.D.); kmorgan3@utk.edu (K.H.M.)

**Keywords:** gut microbiota, early-life gut microbiota, gut dysbiosis, long-term health and disease, Developmental Origins of Health and Disease (DOHaD)

## Abstract

Early life gut microbiota have been increasingly recognized as major contributors to short and/or long-term human health and diseases. Numerous studies have demonstrated that human gut microbial colonization begins at birth, but continues to develop a succession of taxonomic abundances for two to three years until the gut microbiota reaches adult-like diversity and proportions. Several factors, including gestational age (GA), delivery mode, birth weight, feeding types, antibiotic exposure, maternal microbiome, and diet, influence the diversity, abundance, and function of early life gut microbiota. Gut microbial life is essential for assisting with the digestion of food substances to release nutrients, exerting control over pathogens, stimulating or modulating the immune system, and influencing many systems such as the liver, brain, and endocrine system. Microbial metabolites play multiple roles in these interactions. Furthermore, studies provide evidence supporting that imbalances of the gut microbiota in early life, referred to as dysbiosis, are associated with specific childhood or adult disease outcomes, such as asthma, atopic dermatitis, diabetes, allergic diseases, obesity, cardiovascular diseases (CVD), and neurological disorders. These findings support that the human gut microbiota may play a fundamental role in the risk of acquiring diseases that may be programmed during early life. In fact, it is critical to explore the role of the human gut microbiota in early life.

## 1. Introduction

### Developmental Origins of Health and Disease

The microbiome refers to all sequenced DNA in a niche, which includes both living and dead microbes, while microbiota refers to the actual living organisms. The establishment of the signature core microbiome begins in early life and is associated with both maternal pregnancy-related factors and early life events, such as type of delivery, formula feeding, gestational age (GA), adverse childhood events, antibiotic exposure, and ecological factors [1,2,3]. These alterations may set the stage for potential lifelong perturbations in the core microbiome that predict pathophysiology associated with senescence, such as inflammation, insulin resistance, immune senescence, mutation accumulation, and epigenetic changes. There is evidence that the microbiome evolves across the life span, and there are dramatic changes with aging and frailty [4]. Correlative factors associated with these age-related changes include residence, diet, and the degree of retention of the core microbiome that exists, but these are correlated, not causative [5]. Little is known about mechanistic changes in the aging gut microbiota. The immune system becomes educated and expanded in infancy and early childhood, in large part through interactions with the gut microbiota, so alterations in the early gut microbiota could influence later health through retention of early taxa, epigenetics, and immune effects [6,7]. The Developmental Origins of Health and Disease (DOHaD) posits that critical periods in fetal and early childhood environments, which affect growth, metabolism, and neurogenesis, are followed by later environments that determine risk for cardiovascular disease. Like the DOHaD, early-life critical periods have been suggested for the microbiome [8]. There is crosstalk between the gut microbiota and immune development, metabolism, neurogenesis, gastrointestinal integrity, and many other systems across the lifespan, beginning in fetal life. The DOHaD proposes epigenetic variations in infant programming through environmental exposures during critical windows of early life [8,9]. Epigenetic marks allow flexibility of response to environmental signals which affect gene expression levels. Epigenetic reprogramming in mammalian development can occur during critical periods for human development (before fertilization, during embryogenesis, and during a window of plasticity in the first 1000 days of life) [10,11]. Epigenetic programming can be generational, thus preserving the effects of environmental genetic perturbations on offspring [10,12].

The term DOHaD was originally named the Barker hypothesis (1994), which predicted that fetal malnutrition is associated with later adult morbidity and mortality due to chronic diseases [9]. A common finding was that low infant weight was associated with coronary heart disease later in life [8]. In animal studies, maternal malnutrition affected growth and induced lifelong changes in hormonal concentrations which could lead to abnormal organ development. Gluckman and colleagues (2005) introduced the concept of “predictive adaptive responses”, in which environmental factors in early life modify developmental plasticity [13]. The DOHaD hypothesis has since been expanded to account for many types of early life exposures and birth outcomes associated with long-term health and development, including allergic disease, cardiovascular disease (CVD), obesity, diabetes, and neurological disorders in later life [14]. The early gut microbiota may be a modifier of these DOHaD relationships. Colonization of the fetus is minimal or absent, but rapid assembly of the infant gut microbiota is acquired through contact with microbes from the mother and the surrounding environment during and after birth [15]. Sensitive windows in infant development parallel sensitive periods of microbiome development during early life, and the gut microbiota and its associated metabolites may mediate many of the effects on later health and disease.

Gut dysbiosis (imbalances in the gut microbiome abundances caused by the loss of commensals, reduced diversity, or expansion of virulent populations) can impact a broad range of conditions including inflammatory bowel diseases (IBD), atherosclerosis, cancer, metabolic disorders, allergies, autism, and neurodegenerative diseases. The microbiome acquired in the first three years of life remains fairly constant throughout adult life until old age. The phyla abundances are relatively constant, although genera differ with *Faecalibacterium, Dialister, Roseburia, Ruminococcus,* and *Bifidobacterium* at a higher frequency in children compared to adults [16]. Taxonomic signatures found in the infant gut microbiota may potentially remain for years as colonies in the child and adult microbiome [17]. Connecting the development of the early life microbiota to later health and chronic disease is an important area of investigation.

In this review, we have focused on the early life microbiota and its association with human diseases at a later stage of life. Considering the focus on the early life microbiome, we have provided a brief account of it and the factors influencing it. Next, we have summarized the interaction of the gut microbiome with the host immune and nervous system and finally, we have illustrated the association of the gut microbiome for several human diseases based upon the latest knowledge and understanding.

## 2. Diversity and Abundance of the Early Gut Microbiota

**Figure 1:**
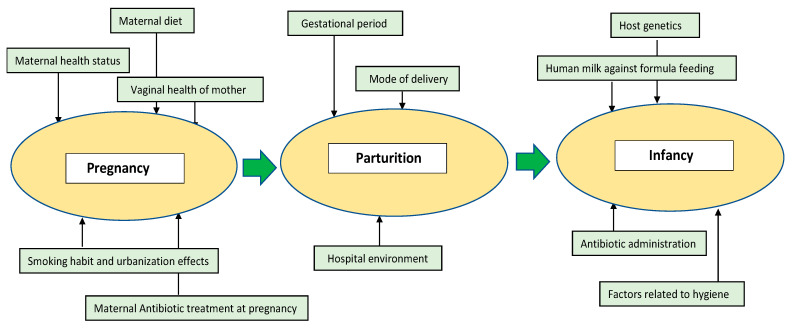
Important factors which affect the microbiome’s richness and abundance at the early stages of life. The early stage of life can be segregated into mother’s pregnancy, parturition, and infancy stage. At each stage, several factors might influence the gut microbiota of the infant including maternal health and habits, local environment, host genetics, and administration of medicines such as antibiotics.

The number of microbial organisms exceeds human cells by a ratio of 1:1.3 [18] and is believed to affect the host’s health throughout life. The microbial colonization of the human body begins at birth, since the uterus is generally perceived to be sterile. However, a few studies have challenged this notion by identifying bacteria from the placenta, meconium, or amniotic fluid, although these claims are considered to be controversial [19,20,21,22]. Sterility is lost after rupture of the amniotic sac, as the baby undergoes the first stage of microbial colonization while descending down the birth canal [23]. Post parturition, there are rapid transformations in the microbiome composition until around three years of age, when the microbiome resembles the diversity and complexity of the adult stage [24,25,26]. As we discuss below, numerous factors influence the diversity and the abundance of the early life gut microbiota in infants. They can broadly be classified into three stages: (i) Pregnancy, (ii) Parturition, and (iii) Infancy. The main factors are diagrammatically represented as Figure 1.

### 2.1. Pregnancy

Several factors during pregnancy can affect the microbial colonization of the infant.

#### 2.1.1. Maternal Health Status

During pregnancy and later, during delivery and lactation, the mother shares her microbes and microbial metabolites with the infant, which highlights the importance of maternal health during pregnancy [27]. It has been reported that high maternal Body Mass Index (BMI) increases the vulnerability of the offspring towards obesity and diabetes later in life due to changes in maternal microbiome composition [27,28,29]. Increased stool levels of *Bacteroides*, *Clostridium*, and *Staphylococcus* were associated with higher BMI and obesity in mothers while *Bifidobacterium* was considerably decreased [30]. Additionally, the levels of *Akkermansia muciniphila*, *Staphylococcus*, and *Clostridium difficile* were decreased in infants of mothers with normal BMI during pregnancy. Maternal gut metabolites may reach the developing embryo and fetus during pregnancy. In a recent study involving mice [31], short chain fatty acids (SCFAs) originating from the gut crossed the placenta and mediated embryonic organ differentiation and metabolism, acting through G-protein coupled receptor (GPCRs) signaling.

#### 2.1.2. Maternal Diet

Gut microbiota composition during pregnancy varies according to the mother’s diet and health status and hence, maternal diet can potentially affect the fetus. The effect of diet on gut microbiota is well known and dietary modulations have resulted in altered gut microbiota in pregnant women [32]. High fat-fed female pregnant mice have been found to gain *Akkermansia* and *Bifidobacterium*, thereby potentially obtaining an altered gene abundance catalogue which can ultimately affect the microbial composition of the infant [33]. The effect of maternal diet on the microbiome was also observed in a primate model (Japanese macaque), affecting the neonatal gut microbiota or inducing dysbiosis [34]. In a separate study on mice, the combination of ethanol-saccharin was found to lower the levels of *Clostridium* while increasing the levels of *Eubacteria* in pregnant mothers [35].

#### 2.1.3. Vaginal Health of the Mother

The vaginal health of the mother is thought to influence the infant’s microbiome because of the direct contact with the fetus during birth. The normal vaginal microbiome primarily consists of *Lactobacilliales*, *Bacteroidales*, and *Clostridiales* [27,36]. Problems in reproductive outcomes such as preterm birth have often been associated with vaginal dysbiosis [37,38]. The comparison between the vaginal microbiota of pregnant and non-pregnant women suggests lower richness and diversity in pregnancy and a lower prevalence of *Mycoplasma* and *Ureoplasma* [39]. *Lactobacillus*, the main component of the vaginal microbiota, maintains a healthy environment and prevents the growth of pathogens by maintaining lower pH and producing bacteriocin [36]. In general, the relative abundance of *Lactobacillus* spp., such as *L*. *crispatus*, *L. gasserii,* and *L. vaginalis*, increases while a decrease in anaerobes such as *Atopobium*, *Prevotella*, *Ruminococcaceae*, and *Parvimonas* across pregnancy has been reported [40,41].

#### 2.1.4. Smoking Habit and Urbanization Effects

Cohort studies suggest that infants exposed to smoke during pregnancy or postnatally have increased gut bacterial diversity of *Firmicutes* at 3 months of age, and increased *Bacteroides* and *Staphylococcus* abundances at 6 months of age [42]. Effects of urbanization and local environment also have impacts on maternal health at pregnancy and postpartum which affects the infant’s gut microbiota. Urban lifestyle, low socioeconomic status, and long working hours lead to pregnant women suffering from “hidden hunger”, micronutrient deficiencies which contribute to malnutrition and can potentially affect the infant [43]. Maternal medications during pregnancy are yet another factor which can potentially affect maternal health and an infant’s gut microbiota [43].

#### 2.1.5. Antibiotic Treatment at Pregnancy

Antibiotic treatment at pregnancy can strongly affect the maternal and infant microbiome which, in turn, can affect the development of the immune system of the infant [44,45]. Both maternal and infant gut microbiota composition undergo noticeable changes with *Streptococcus* dominating the GI tract in mothers, while in infants, *Enterococcus faecalis* becomes predominant in treatment with antibiotics at pregnancy and during lactation [44]. A reduced microbiome diversity has also been found in infant mice when mothers were treated with antibiotics during pregnancy [46]. A major source of antibiotic treatment at pregnancy occurs in Cesarean section (CS) which is quite common in western countries [23]. Even in vaginal delivery, the time of antibiotic administration is important and is associated with an increase in *Clostridium* and reduction in *Bifidobacterium* [47].

### 2.2. Parturition

#### 2.2.1. Gestational Age

Depending on the gestational age (GA), infants can be classified into term (GA: 37–42 weeks) and preterm infants (<37 weeks) [48]. Infants who are born prematurely have lower gut bacterial diversity and often reduced *Bifidobacterium* [49]. A recent study involving 45 preterm infants found that GA contributed the most to infant gut microbiota development, although the infants tended to catch up with the normal term infants over time [50]. In another study involving 65 stool samples from preterm infants and 189 samples from term infants, the importance of GA along with the absence of many important bacterial genera in preterm infants was observed [51]. Even though preterm infants slowly acquire the diversity of the normal term infants, 25% of such infants experience neurodevelopment problems [52]. In addition, the problem is exacerbated as they are more likely to receive antibiotics during hospitalization, leading to dramatic changes in their gut microbiota.

#### 2.2.2. Mode of Delivery

The mode of delivery is the most important influence on the initial neonatal gut microbiota. The impact of delivery mode lasts for months and probably longer, which in turn can affect normal metabolism, health status, and vulnerability towards various diseases [27]. After a vaginal delivery, the neonate first encounters the vaginal and the gastrointestinal (GI) tract microbiota of the mother, which predicts the first gut microbiota. Cesarean section (CS) delivery does not allow the neonate to encounter the vaginal and enteric microbiota and thus the infant acquires microbiota from the mother’s skin and the hospital surroundings.

#### 2.2.3. Hospital Environment

The hospital nursery and neonatal intensive care unit (NICU) environments, cohabitation with family members and the presence of hospital staff, geographical location, and air quality together comprise the hospital environment which might affect the neonatal gut microbiota. Even though the NICU environment is sanitized regularly, stubborn pathogenic and commensal bacterial species are found within these facilities [23]. *Neisseria*, *Staphylococcus*, *Streptococcus*, and *Enterobacteriaceae* species have been found frequently on the surfaces of medical equipment such as feeding tubes, catheters, stethoscopes, and pacifiers [53,54]. On the other hand, *Halomonas, Gemella*, and *Acinetobacter* species comprise the major environmental bacteria inside the NICU facility [23,54,55]. The exact route by which the environmental microbes are transferred to the infants is difficult to ascertain, but specific microbes from the local surrounding have been previously isolated from the infant fecal samples [25]. In a different study, the *Klebsiella* species, which often harbors antibiotic resistant genes, was hypothesized to enter into the infant gut from the hospital environment [56].

### 2.3. Infancy

#### 2.3.1. Human Milk vs. Formula Feeding

Breastfeeding constitutes the first dietary exposure to the newborn from the mother. This process also shapes the gut microbiota in the newborn. The neonate is exposed to the milk microbiome and secondly, maternal milk factors, such as the human milk oligosaccharides (HMOs), anti-microbial factors, and secretory IgA (SIgA). WHO recommends that infants should be exclusively breastfed for the first six months of life. To understand the effect of feeding on newborns, several studies have been conducted to compare the effects of human milk compared to formula milk on the infant microbiome. Although *Bifidobacteria* represent the most abundant bacteria in both human and formula milk, breastfed infants have a more uniform and stable microbiota [57,58,59]. Human milk has also been found to provide resistance against diarrhea, necrotizing colitis, allergy, and coeliac disease [60,61,62].

#### 2.3.2. Host Genetics

Host genetics are important factors which interact, modify, and regulate the host microbiome at every stage. Several studies have identified associations between host genetics and microbiome [63,64,65]. Studies involving unrelated controls and twins have suggested that the sharing of microbiome is higher in identical twins followed by non-identical ones and the lowest in unrelated individuals, suggesting some degree of host control over the microbiome composition [66]. In addition, several genome variants in the genes IRGM2, CARD9, and NOD2 are linked to IBD and the genus *Roseburia* is associated with increased risk of IBD in healthy controls [67]. It has also been shown that *Christensenella* from the gut is heritable and 40% of its variance can be explained by genetic factors [68]. Although the roles and controls of host genetics over microbial colonization have been repeatedly reported, their mechanisms are yet to be fully understood.

#### 2.3.3. Antibiotic Administration

Antibiotic administration, as expected, results in considerable changes in the infants’ gut microbiota. If the antibiotic treatment is given at an earlier stage of infancy, it affects the microbiome to a greater extent. Similarly, more frequent administration causes more damage to the microbiome and leads to lesser production of antibacterial compounds and IgG, making the infants susceptible to infections [69,70]. Antibiotic resistant microbes might become pathogenic at some stage. In such cases, the metabolism of SCFAs might be altered and be associated with elevated levels of inflammatory cytokines, leading to immune-mediated diseases including diabetes and asthma [69]. If antibiotics are administered at an infant stage, it increases vulnerability towards obesity and IBD [71]. Antibiotic resistance is a very serious issue all over the world with indiscriminate use of antibiotics consistently building up the resistome profile [72].

#### 2.3.4. Factors Related to Hygiene

The hygiene hypothesis proposed by Strachan emphasizes the relationship of exposure of the immune system in infants and children towards microbes and subsequent health. Excessive hygiene leads to an underdeveloped immune system which fails to gain memory and thus, lacks the preparedness to fight infections in future encounters [73]. Environmental factors related to hygiene such as growing up on a farm and low socioeconomic lifestyles have been associated with greater exposure to infections and subsequent reduced incidences of allergies and autoimmune diseases. Developing countries with lower incomes often have lower prevalence of allergic infections, even at a very early stage of life [74]. The presence of older siblings and higher number of family members leads to appropriate alterations in the infant’s gut microbiota, enabling the ability to fight infections and reducing risk of allergies at a later stage of life [75]. The role of the commensal gut microbiota in these relationships remains to be explored, but dysbiosis is frequently noted in patients with autoimmune disease.

## 3. Early Gut Microbiota and Immunity

Support is growing for the idea that infant and early childhood microbial colonization and succession are important for subsequent maturation of the immune system [76]. The infant immune system differs from the mature adult immune system, and in particular, preterm infants are susceptible to infections [77]. During the last decade, evidence has accumulated to support the theory of a critical window for both gut colonization and immune development [78,79,80]. Environmental factors after birth, such as hospitalization, antibiotic usage, or diet, further modulate the development of the infant’s microbiome and immune system. Exposure to a variety of microbial organisms during early life appears to exert a protective effect [81,82]. The commensal bacteria may be involved in the development of lymphoid structures in the GI tract, such as Peyer’s patches and isolated lymphoid follicles, the promotion of intestinal epithelial cells (IECs), and the development of GI mucosal angiogenesis [83,84,85]. The early gut microbiota maintains a state of regulated inflammation that provides the cross talk between gut-associated lymphoid tissue (GALT) and gut microbiota which influences the education of the adaptive immune system [86,87]. The interaction between IECs and lymphoid tissue and the colonization of commensal microbiota are essential for the development of a normal infant gut and immune system. Commensal gut bacteria contribute to induction of apoptosis, reactive oxygen (ROS) synthesis, and Toll-like receptor (TLR) signaling that help to develop innate immune defenses and promote recognition of pathogens [88,89]. Evidence that the host immune system depends on microbial gut colonization is that antigen-specific secretory IgA (SIgA) is produced by the gut-associated lymphoid tissues (GALT). SIgA is the major mucosal antibody in the gut with the role of neutralization of bacterial toxins in the gut lumen [90,91]. For example, rotavirus infection is neutralized throughout the epithelial barrier and sIgA inhibits its epithelial translocation and inflammatory potential [92]. Planer and colleagues (2016) also showed that the development of gut microbiota in infants affected the progression of intestinal mucosal SIgA responses in 40 healthy twin pairs infants [93]. Lack of SIgA responses leads to gut microbial dysbiosis, which can produce immune hyper-activity. The interaction between commensal bacteria and host IECs and immune cells plays a pivotal role in the development and balance of the immune system [83,84].

### Gut Dysbiosis in Early Life and the Immune System

Dysbiosis is a term used to describe alterations in the balance of commensal to virulent microbes, changes in diversity, and inflammatory potential that may lead to increased gut permeability. Early gut dysbiosis is associated with increased abundances of potential pathogenic microbiota. Changes in bacteria composition and diversity can lead to disruption in the epithelial barrier and increase susceptibility to infections and immune disorders [94,95,96]. A large longitudinal human cohort study (100 newborn infants) found that the first 3 months of life were significant as microbial dysbiosis during this period was associated with immune system development. Infants with early gut dysbiosis had increased circulating endothelial cells, activation of T cell populations, and higher levels of a pancreatic digestive enzyme in their 3-month blood samples [77]. *Bacilli* or *Gammaproteobacteria* were predominant in these infants with gut dysbiosis, which would impact the developing immune system. Malnutrition may lead to microbial dysbiosis, which can increase the risk for inflammation and permeability in the gut. Impaired immunity and changes in gut microbiota have been strongly implicated in childhood malnutrition. Both animal and human studies showed that infant gut dysbiosis and metabolic dysfunction contribute to the risk of childhood atopy and asthma [97,98,99]. CD4+ T cell dysfunction may be associated with gut microbial dysbiosis and be etiological in atopy [99]. Furthermore, early-life microbiome disruption due to CS, antibiotics usage, or other possible factors are associated with increased risk of overweight status later in childhood [100,101,102]. However, it has been argued that the evidence is not convincing for an association between antibiotic exposures in early life and an increased risk of overweight and obesity in later childhood [103].

## 4. The Microbiome–Gut–Brain Axis

Brain growth and development during the first 3 years of life occur in parallel with development of the gut microbiome. The gut microbiota produces neuroactive compounds, such as peptides and mediators, which develop a bidirectional communication with the brain through the neural network, endocrine system, and inflammatory pathway. The neuroactive compounds are microbial-derived intermediates that can influence neuronal functions and behavior [104,105]. Microbial-derived SCFAs promote microglial maturity and proper functioning. Microglia function as macrophage-like cells of the brain, comprising 10–15% of all glial cells [104]. Microglia are the major innate immune cell type in the Central Nervous System (CNS), and play roles in early brain development, antigen presentation, phagocytosis, and modulation of neuroinflammation throughout life [104]. These early gut–brain effects on the CNS may set the stage for later neurological or neuropsychiatric disease.

## 5. Relationships between the Early Gut Microbiome and Later Disease

**Figure 2:**
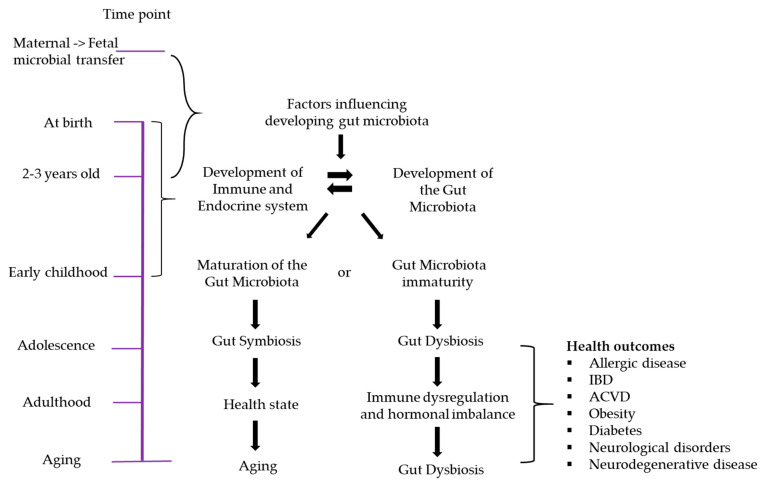
The relationship between lifespan and development of the gut microbiota. Many factors influence gut microbiota development, and certain critical time points in life are associated with the maturation of both the gut microbiota and the immune system. At infancy, if dysbiosis occurs due to factors such as prematurity, CS delivery, NICU admission, antibiotic usage, etc., the development and lifelong functions of many host physiological systems could be affected, leading to disease.

Over the past decade, there has been a plethora of evidence that the gut microbiota modulates many physiological, metabolic, and immunological functions. Thus, the influence of the early-life gut microbiota is the focus of intense research. Here, we discuss the potential association of childhood and adult diseases with the early gut microbiota and gut metabolites. (see Figure 2).

### 5.1. Allergic Diseases

Low gut microbial diversity and microbial colonization are associated with the developing infants’ immune system and may contribute to immune-mediated diseases such as asthma or atopic dermatitis. Colonization of the infant gut with *Escherichia coli* may be associated with increased risk of eczema [106]. Atopic dermatitis is a chronic and relapsing skin disorder that is diagnosed during early infancy, childhood, or adolescence and is often associated with food allergies. It may disappear as children get older, but other atopic diseases such as asthma or allergic rhinitis may follow in adulthood. Atopic dermatitis is often associated with elevated serum IgE levels and a family history of at least one of the atopic diseases such as food allergy, allergic rhinitis, and asthma [107]. In the gut, IgE is produced in response to food allergens and serves as an indicator of food sensitization.

There is a relationship of dysbiosis of the early gut microbiome and asthma, and asthma may continue to be present in adult life. Asthma is seen more frequently in children who were born by CS or who received multiple doses of antibiotics in early life [108]. Colonization of the infant gut at one month of age with *Clostridium difficile* was associated with later asthma at 6 to 7 years of age [109]. *Proteobacteria* in the gut microbiota is a dominant phylum in children with asthma.

### 5.2. Obesity

It has been recognized that childhood and adult obesity are associated with prenatal and infant growth patterns. Evidence suggests that excessive weight gain during the first two years of life is significantly associated with later obesity [110]. Additionally, adults born with low birth weight have been identified to have higher risk of impaired glucose tolerance, metabolic syndrome, and cardiovascular diseases compared to adults who were born average weight [111,112]. One possible hypothesis pertaining to the increased risk factors could be growth restriction during the fetal period. Early growth restriction is associated with a heightened adiposity in childhood and later adult life [113] via alteration of insulin sensitivity [114]. Low birth weight individuals tend to develop central adiposity which predicts later metabolic disorders [113].

The widespread use of antibiotics in the treatment of infectious diseases, as well as the exposure humans have to antibiotics through agriculture, can contribute to the development of obesity. The gut microbiota composition is thought to play a role in the development of obesity [115]. Antibiotics in both humans and animals change the gut microbiota composition towards an obesogenic profile [116]. These adverse effects of antibiotics need to be studied longitudinally, especially in vulnerable populations such as pregnant women, infants, and children. Gastrointestinal function and nutrient digestion and absorption are affected by both the microbiome and multiple gastrointestinal hormones, enzymes, and microbial metabolites. For example, gut-derived metabolites, such as SCFAs, bind and activate GPR41 which results in the expression of peptide YY. Stimulated peptide YY expression suppresses intestinal transit, enhances hepatic lipogenesis, and expands energy harvests from nutrients [117]. SCFAs also have many effects on the intestinal barrier, colonocyte health, and inflammation.

The ratio between *Firmicutes* and *Bacteroidetes* is associated with energy extraction from non-digestible food and fat storage. Moreover, the composition of the gut microbiota is strongly related to weight. A longitudinal study has demonstrated that in average weighing 7-year-old children, there were more *Bifidobacterium* in their fecal samples during infancy. In contrast, overweight children presented a higher number of *Staphylococcus aureus* in their infancy stool sample [118]. Other studies also support that the number of *Bifidobacterium* was inversely correlated with glucose intolerance, BMI, and lipopolysaccharides (LPS) level [119,120]. Furthermore, a large longitudinal cohort study demonstrated that the abundance of *Bacteroides fragilis* at one month of age in 909 infants was significantly associated with higher BMI z-scores in children up to 10 years of age [121].

The interaction between low-grade inflammation and gut microbiota is one of the main contributors for increasing levels of obesity in both children and adults. For example, Cani and colleagues (2007) indicated that both metabolic inflammation and circulation of plasma LPS concentration were increased in high-fat diets. This leads to enhanced insulin resistance and adiposity [122]. Scientists also identified that depletion of LPS (CD14 knockout mice) [122], and TLR4 knockout mice, are resistant to diet-induced obesity and hepatic insulin resistance [123]. The function of gut microbiota in low-grade inflammation has also been supported by a study in which inflammation did not develop in germ-free mice when a high-fat diet was provided [124]. The expression of serum amyloid A3 protein (SAA3) is known to provoke inflammation, accumulate macrophages in adipose tissue, and increase weight gain in female mice on high fat diets, whereas SAA3 knockout mice gained less weight [125]. These data suggest that modifying the gut microbiota composition during early life could play a key role in limiting the global obesity pandemic.

### 5.3. Atherosclerotic Cardiovascular Diseases (ACVD)

A number of studies support that certain conditions in infancy, including malnutrition, preterm birth, or colonization of gut microbiota, can predispose an individual to developing ACVD in adulthood [8,111,112,126]. The gut microbiota composition is strongly influenced by neonatal and childhood malnutrition [48,127]. A reduction in commensal gut bacteria, especially *Bifidobacterium*, may lead to inefficient digestion. Decreased utilization of dietary carbohydrates and reduced vitamin production could lead to malnutrition [126]. Moreover, increased levels of *Proteobacteria*, a potentially pathogenic bacterium in preterm infants, has been found in some adults with ACVD [112,128]. An increase in pathogenic taxa is not only responsible for reduced nutrient absorption but can also cause epithelial damage and increase inflammation. This epithelial damage can lead to reduced gut barrier permeability. Systemic circulation of bacteria and metabolic products ensues, leading to increased systemic inflammation, an underlying cause of ACVD [129,130]. As mentioned earlier, the effects of gut metabolites and gut dysbiosis strongly suggest that the gut microbiota plays a significant role in contributing to ACVD via increased inflammation or control of cholesterol metabolism. It is well known that the atherosclerotic process begins early in life with the formation of the fatty streak. Diet is believed to be important, but of course diet also influences the gut microbiome. ACVD is a chronic inflammatory disease in which lipids accumulate in the artery wall while the vascular smooth muscle cells form a fibrous collagenous cap, commonly referred to as a plaque, that is then infiltrated by immune cells, including macrophages, T cells, and mast cells [131]. This atherosclerotic process is the most common underlying cause of ACVD and is associated with a wide variety of risk factors, including hypertension, diabetes, low-grade inflammation, and alteration of gut microbiota [132]. As an example of their direct involvement, gut bacteria have been found within atherosclerotic plaques [133] and may contribute to the initiation of atherogenesis. Gram-negative bacteria can stimulate platelet aggregation, thrombus formation, or act through their structural components, including LPS, to trigger an inflammatory cascade, increasing the expression of IL-1β. These effects contribute to the vascular and coagulation processes involved in atherosclerosis [134]. Additionally, bacteria present in the plaques correlate with clinical findings such as levels of cholesterol, alanine aminotransferase, and fibrinogen [133]. However, numerous studies have also explored several ways the gut microbiota indirectly influences ACVD development via its metabolites (SCFAs, trimethylamine, trimethylamine-N-oxide, and bile acids), controlling host systemic inflammation, activating the innate immune system, and directing the adaptive immune response. These metabolites can act as signaling molecules that bind to specific receptors on distant organs or can act even more indirectly by interacting with other endocrine molecules. When SCFA production is suppressed, there can be severe consequences including intestinal inflammation, decreased protection against pathogen invasion, reduced gut barrier integrity [135], loss of immune tolerance [136,137,138], dysregulation of liver cholesterol synthesis [139], and reduced atherosclerotic plaque stability and macrophage polarization towards a pro-inflammatory phenotype [140].

### 5.4. Diabetes

Diabetes is considered the epidemic of the century [141]. Diabetes represents a group of metabolic diseases characterized by chronic hyperglycemia due to dysfunction in insulin secretion, function, or a combination of both. Metabolic abnormalities in carbohydrates, lipids, and proteins result from the importance of insulin as an anabolic hormone. The severity of symptoms can be attributed to the type and duration of diabetes. Diabetes mellitus can be classified into three types: Type 1, Type 2, and Gestational Diabetes mellitus (GDM). Type 1 diabetes (T1DM) is autoimmune and results in the immune destruction of insulin-secreting β cells of the pancreas. Type 2 diabetes (T2DM) is characterized by insulin resistance; affected individuals cannot utilize insulin. Gestational diabetes occurs during pregnancy and is associated with hormonal changes, genetics, and lifestyle.

Infants delivered by CS were found to be at a higher risk for childhood T1DM [142]. CS delivery is associated with an altered gut microbiota acquisition and a less trained immune system, potentially increasing susceptibility towards T1DM. Progression to diabetes in rodents has been associated with altered microbiome composition, reduced abundance of *Firmicutes* compared to *Bacteroidetes*, and a reduction in butyrate-producing bacteria [143]. The DIABIMMUNE study, which was aimed at exploring relationships between the hygiene hypothesis and T1DM, recruited infants from three countries (Estonia, Finland, and Russia) who had a human lymphocyte antigen (HLA) predisposition for autoimmune disorders [144,145]. The children who developed T1DM experienced a clear drop in alpha diversity and there was an increased abundance of *Blautia*, *Rikennellaceae*, *Ruminococcus*, and *Streptococcus*, while *Coprococcus eutactus* and *Dialister invisus* were absent. The effect of geography is also to be considered, as shown in the TEDDY study which incorporated infants from four countries (Finland, Germany, Sweden, and USA) [146]. The children in Finland and USA had considerably reduced bacterial diversity while *Bifidobacterium* was higher in USA and Sweden compared to other locations. Similarly, another study involving 33 infants predisposed to T1DM reported reduced bacterial diversity and a spike in inflammation-promoting bacterial species such as *Ruminococcus gnavus* and *Streptococcus infantarius* [147]. Additionally, the authors found elevated levels of human b-defensin 2 (hBD2) in those children who later develop T1DM. hBD2 is a known antimicrobial product produced by colonic epithelial cells during inflammation [148]. Considering the importance of gut microbiota in infancy and T1DM, they administered the probiotic *Escherichia coli Nissle* 1917 (EcN) strain to 54 newborn infants for the initial five days of life [149]. This strain remained detectable after six months in the infants and levels of pathogenic bacteria were reduced compared to the placebo group. *EcN* is associated with increased IL-10 in vitro while it reduces the secretion of TNF-alpha from peripheral T cells, indicating its anti-inflammatory effect [150,151].

### 5.5. Inflammatory Bowel Disease (IBD)

IBD disorders are hyperimmune, multifactorial disorders and include Crohn’s disease and ulcerative colitis. Both disorders are associated with inflammation and alterations in the gut microbiota and metabolome and have strong genetic underpinnings. Genes known to be associated with IBD are risk factors for particular microbiota composition even in healthy individuals [67]. A lower abundance of *Roseburia* and decreased microbial diversity in patients have been noted [67]. These diseases can first appear in childhood and adolescence, and present with lifelong chronic, relapsing courses. Since education and shaping of the immune system occurs in early life, the contribution of the infant and early childhood microbiome and metabolome may be potentially important in disease development. Exposure to antibiotics during fetal life, but not in infancy, has been associated with IBD development in childhood [152]. Crohn’s disease course in children was influenced by environmental factors, such as breastfeeding and exposure to cigarette smoke [153]. Infants born to mothers with IBD showed increased abundances of *Gammaproteobacteria* and reduced *Bifidobacteria* through the first three months of life [154]. It is not clear if these relationships are causative or correlative, as the chronic inflammation of IBD could impact gut microbiota rather than dysbiosis causing IBD [155].

### 5.6. Neurological Disorders

#### 5.6.1. Neuropsychiatric Disorders

A disrupted microbiome may be associated with the development of psychopathology, such as mood and affect disorders (anxiety and depression), schizophrenia, bipolar disorder, Parkinson’s disease, Autism Spectrum Disorder (ASD), and Attention-deficit/hyperactivity disorder (ADHD) [156,157,158,159,160,161,162,163]. Several brain signaling pathways, including the hypothalamus–pituitary–adrenal axis (HPA), immune pathways, and microbial- driven neuroactive compound pathways, connect with the gut microbiota. The gut microbiota generates a profound effect on HPA axis development [164] and epigenetic modulation [165], contributing to future maladaptation of the stress response and increasing the risk of mental health difficulties in adulthood [166]. This happens because early adverse/stressful events can produce long-term epigenetic changes that affect the HPA axis as well as neurotransmitter and neuroendocrine signaling, which may then impair adult cognitive and behavioral functioning [156]. Moreover, altered levels of microbiome-produced metabolites may induce modifications of key disease-susceptible gene expression during critical periods of neurodevelopmental disorders [167].

The hypothalamus–pituitary–adrenal (HPA) axis can be (re)programmed in early life [10] and is a neuroendocrine system that is involved in stress responsivity, immune function, and metabolic efficiency. The cortisol produced by the stress responsive HPA helps the organism to adapt to increased demands and maintain health by mobilizing resources (carbohydrates, fats, and proteins) to provide energy. The HPA also interacts with the immune, sympathetic, neurological, and cardiovascular systems [168]. Stress exposure may magnify pathophysiological effects in multiple systems.

Germ-free mice, which lack any microbiota, have impaired social behavior, reduced anxiety, and abnormal stress responses. Certain behaviors in germ-free animals are correlated with neurochemical changes in the brain [169]. Germ-free mice exhibited higher adreno-corticotropic hormone (ACTH) and corticosterone release after mild restraint stress compared to controls. However, this exaggerated stress response was reversed by giving the mice *Bifidobacterium infantis* at an early stage, which suggests that there is a critical time for colonization of the gut for normal HPA axis development [170]. Stress also impacts the microbiome composition [171]. HPA axis dysfunction is associated with many psychosomatic and psychiatric disorders.

#### 5.6.2. Neurodegenerative Diseases

While longitudinal studies in humans are markedly absent, a growing body of evidence from cross-sectional studies and animal models demonstrate correlative relationships between neurodegenerative diseases (NDDs) originating via the microbiome–gut–brain axis, especially when the gut is dysbiotic. Elucidating the functional mechanisms for these correlations, and possible causal relationships, will allow the identification of targets for early diagnosis, intervention, therapeutics, and possibly prevention [172]. As previously noted, brain development and maturation in infancy and early childhood may be disrupted by gut dysbiosis. This may produce a brain more vulnerable to later life neurodegeneration.

Dysbiosis commonly occurs as adult humans age into their elder years (see Figure 2). Dysbiosis with aging could occur as a result of any one or combination of the following factors occurring across the lifespan from early childhood onwards, such as oligotypes retained from dysbiosis in infancy or childhood, environmental changes, antibiotic and other medication use, hormonal imbalances, metabolic or other disease states, epigenetics, or a combination of any of these. Dysbiosis can set up the gastrointestinal tract to “seed” the CNS through a leaky mucosal lining, leading to exposure to pathogens, low molecular weight metabolites or other byproducts through the blood–brain barrier (BBB), or via the enteric nervous system (ENS) and the vagus nerve. The ENS governs complex physiological regulations of the gastrointestinal tract such as peristalsis, and is integral to the function of the gut; it is exposed to the microbiome and nutrients, and communicates with the gut immune system [173]. The ENS contains neurons and glial cells distributed throughout the wall of the gut [174]. It relays messages between the gut and the central nervous system (CNS) along the vagal parasympathetic and sympathetic tracts. Maturation of the ENS in germ-free (GF) mice is improved by colonization with microbiota from conventionally raised mice, demonstrating a complex interplay between the microbiome and its role in the development of ENS function in an animal model [175].

There are several plausible pathways for a microbiome–gut–brain hypothesis for the origins of NDDs. A few examples are found in Parkinson’s Disease (PD), Alzheimer’s Disease (AD), and Amyotrophic Lateral Sclerosis (ALS).

##### Parkinson’s Disease

Parkinson’s disease (PD) is the second most prevalent neurodegenerative disease (NDD) after Alzheimer’s disease (AD) [176]. Both are α-synuclein (α-Syn) diseases characterized by amyloid protein deposition [177]. In PD, α-Syn depositions form neuronal inclusions called Lewy bodies, a hallmark of PD. Lewy bodies have been found in the intestinal enteric nerves, which led to the hypothesis that PD may start in the intestine and move to the brain via increased intestinal permeability in the presence of α-Syn [178]. Another of the proposed methods of crosstalk along the gut–brain axis in PD is via the vagus nerve, with the enteric nervous system (ENS) seeding the brain via the vagus nerve [179]. Bidirectional gut–brain crosstalk may play a role in PD development, and persons with PD have increased *Helicobacter pylori* and *Ralstonia* [180]. Several studies implicate gut microbiota, dysbiosis, and PD. Compared to 36 cohabiting healthy controls, 52 PD patients had more lactobacillus, fewer hydrogen-producing bacteria, and lower serum lipopolysaccharide (LPS)-binding protein levels [181]. In another study of gut microbiota of PD versus controls, it was found that the PD cohort had reduced abundances of the phylum *Bacteroidetes* and the family *Prevotellaceae*, while *Enterobacteriaceae* increased; also, fecal short-chain fatty acids (SCFAs) were reduced [182]. Unger et al. propose that the reduced number of SCFAs may account for altered ENS function as one cause of reduced gastric motility.

There is a growing body of evidence for a hypothetical PD etiology beginning in the gut, with microbiota. A recent epidemiological study of 1.6 million individuals found that appendectomy (excision of the appendix) in young adulthood correlated with a lower risk for PD and that the human appendix contains α-Syn and Lewy body products [183]. Some 20 years prior to receiving a clinical diagnosis of PD, a person developing PD usually experiences functional gastrointestinal symptoms, such as constipation, which has been linked to dysbiosis [184]; then years later, a constellation of symptoms such as hand tremor, bradykinesia, muscle rigidity, masked facies, and shuffling gait becomes observable. By the time symptoms of a movement disorder are noticed, the substantia nigra has already lost 60 to 66% of its dopaminergic neurons [185,186], resulting in a loss of dopamine supply through the striatum to the basal ganglia, the thalamus, and motor cortex. A large cohort study of 197 PD patients versus 130 healthy controls found an independent PD 16s rRNA microbial signal after controlling for confounders (medications and diet), altering abundances of families of *Bifidobacteriaceae, Christensenellaceae, Tissierellaceae, Lachnospiraceae, Lactobacillaceae, Pasteurellaceae*, and *Verrucomicrobiaceae*; furthermore, PD medications strongly affect microbial abundance and diversity [187]. Studies such as the above lend credence to the hypothesis that PD begins in the gut.

Zhu et al. [188] recently provided evidence in a PD mouse model that gut dysbiosis is associated with disturbances in the Dopamine, Kynurenine, and 5-hyroxytryptamine metabolic pathways of neurotransmitters involved in the gut–brain axis, increased α-Syn, and increases in other abnormalities associated with PD development. Their results lend evidence that gut microbial dysbiosis is linked to changes in neurotransmitter metabolism along the gut–brain axis, eliciting worsening of PD-related changes such as an increase in apoptotic proteins in the substantia nigra neurons and altering tryptophan and tyrosine protein metabolism in the striatum. Persons with PD have an α-Syn aggregation in the colon, decreases in bacteria with anti-inflammatory properties, such as *Blautia, Coprococcus,* and *Reseburia* species, and colonic inflammation [189]. In a separate study using a GF mouse model overexpressing α-Syn, fecal microbiota from human PD patients and healthy controls were transplanted into GF mice by oral gavage [190]. Mice receiving fecal transplants from PD patients suffered motor deficits and neuroinflammation, leading authors to postulate that “postnatal signaling between the gut and the brain modulates disease” [190]. The same mice improved with antibiotic treatment.

##### Alzheimer’s Disease

Alzheimer’s disease (AD) is the most common NDD and cause of dementia. Like PD, AD pathogenesis is marked by abnormal protein deposition in the brain, namely the overproduction of amyloid beta (Aβ) peptides that form Aβ plaques as well as a second protein, hyperphosphorylated tau, which is insoluble and forms neurofibrillary tangles (NFTs) in the cytoplasm of neurons. Tau appears to be seeded in the brain many years prior to the emergence of observable symptoms, and spreads throughout the brain within 10–20 years [191]. Significantly, genome-wide association studies (GWAS) suggest that signals related to AD are not found within human gene coding regions, which suggests an epigenetic or environmental etiology [192].

To explore the role of microbiota in AD, Vogt et al. [193] characterized the microbial taxa associated with AD by sequencing bacterial 16S rRNA of fecal samples from AD participants and age and sex-matched healthy controls who did not have dementia. Gut microbiota abundances and richness were decreased in AD participants. Specifically, Firmicutes and Bifidobacterium decreased, while Bacteroidetes increased in AD participants’ gut microbiome compared to healthy controls. Using cerebrospinal fluid (CSF) biomarkers to detect the level of AD pathology, microbiota abundances were correlated with biomarkers of AD pathology. Bacterial relative abundance and CSF biomarkers of AD consistently demonstrated correlations with trends consistent across AD and control participants. For example, positive or increased abundance in bacterial genera in AD correlated with increased amyloid burden in the brain as measured by CSF Aβ_42_/Aβ_40_. These changes in gut communities in AD participants support the gut–brain hypothesis influencing the development of neuropathology, from gut to brain. An AD mouse model lends support to a microbial etiology of Aβ pathology [194]: Aβ precursor protein (APP) transgenic mice demonstrate dysbiosis compared to wild-type mice. Additionally, germ-free APP transgenic mice show lower Aβ pathology, compared to controls, and colonization of germ-free APP transgenic mice with microbiota from APP transgenic mice increased Aβ pathology in the brain.

In a study of an oral microbe, *Porphyromonas gingivalis*, which is the primary bacterial cause of periodontitis, Dominy et al. [195] found *P. gingivalis* and its toxic protease, gingipains, in the brains of AD patients by conducting tissue microarrays on AD and sex- and age-matched brain tissue cores, which correlated with tau and ubiquitin pathology. Interestingly, in nondemented control tissues, antibodies indicating gingipain to ubiquitin load and gingipain load and tau load revealed some AD pathology was present in controls preclinically (meaning no observation of clinical signs or symptoms were present in non-demented, healthy controls). *P. gingivalis* DNA was also detected in the CSF of AD patients. In mice, *P. gingivalis* oral infections led to brain contamination with *P. gingivalis*, and mouse brain Aβ 1-42 production increased significantly over controls. These findings lend support to other reports that Aβ 1-42 is an antimicrobial peptide. Furthermore, an inhibitor designed to block gingipain effectively reduced brain bacterial load of *P. gingivalis* in BALB/c mice.

##### Amyotrophic Lateral Sclerosis

Amyotrophic lateral sclerosis (ALS) is a progressive, fatal NDD that initially presents as either upper motor neuron or lower motor neuron weakness, such as loss of hand grasp and coordination or foot drop and spastic gait, respectively. Motor neurons extend from the brain to the spinal cord and out into the peripheral nervous system to innervate the muscles. ALS is characterized by systemic and CNS inflammation and problems in energy metabolism [196]. It is associated with frontotemporal dysfunction, which affects the person’s executive function and behavior among many other symptoms associated with the frontotemporal lobe. The final disease stage usually results in neuromuscular respiratory failure as the most common cause of death [197]. Only a small percentage of ALS cases are genetically linked or familial. The gut–brain axis may play a role in ALS; one hypothesis is that low-molecular-mass metabolites and other translocation of bacterial products originating in the gastrointestinal tract can permeate a leaky gut to cross the BBB to modify neurons, among other types of cells, via epigenetic and transcriptional alterations [198]. Other causes of motor neuron death include oxidative stress (due to mitochondrial dysfunction), excess excitation via glutamate, excess accumulation of the neurotransmitter GABA, proinflammatory cytokines and inflammation consistent with infection, and abnormal astrocytes that become neurotoxic when induced by microglia [199].

Blacher et al. [200] found differences in a mouse model between an ALS cohort of *Sod1* transgenic mice and healthy mice microbiota and metabolome. However, in humans, there have been conflicting results in gut microbiome characterizations of ALS. Brenner et al. [196] conducted an observational cohort study of ALS versus healthy controls using 16S rRNA gene sequencing and predictive metabolome analysis. While the ALS cohort had a higher number of OTUs, there were no significant differences between ALS and control microbiota diversity, abundance, or predictive metagenomes. By contrast, Fang et al. [201] used high throughput sequencing to find significant differences between the ALS cohort and the healthy controls at the level of the genus and also a decreased Firmicutes/Bacteroidetes ratio at the level of the phylum. Furthermore, they found that there was an increase in *Dorea*, a genus associated with increased gut permeability, and a reduction in more beneficial microorganisms including *Oscillibacer, Anaerostipes,* and *Lachnospiraceae*, constituting dysbiosis that could provide a pathogenic etiology of ALS.

More recently, Gotkine et al. identified dysbiosis in an ALS *Sod1* transgenic mouse model and in humans with ALS, and further, *Sod1* transgenic mice motor function was improved by the introduction of *Akkermansia muciniphila*, possibly due to its production of nicotinamide [202]. Another study on the *Sod1* transgenic mouse model also found dysbiosis, epigenetic, and immunological changes prior to the onset of motor deficits [203]. Animal models continue to make progress towards targeting therapies for ALS by experimenting with and intervening in the microbiota along the microbiome–gut–brain axis. In humans, further investigations of the microbiome–gut–brain axis as an etiological pathway and target for interventional treatment in many systems are necessary. Obrenovich et al. [199] outline some of the barriers and questions relative to ALS studies in humans and point towards the success of fecal transplantation as a possible mitigating factor in PD, AD, and ALS, among other suggestions.

## 6. Conclusions and Future Perspectives

Acquisition of gut microbiota and a stable gut microbial community in the early stages of life is closely associated with human health. The development and succession of the microbiome is highly interconnected with the development and education of the immune system, and many of the diseases we have discussed have strong immune and inflammatory etiologies. Infancy and early childhood may represent critical windows for both human development and microbiome successional maturation in many systems. The many influences on the early gut microbiome appear to have both short- and long-term influences on health and diseases. The DOHaD hypothesis is a relevant approach to analyze potential mechanisms by which the early gut microbiome may influence later health independently and in interaction with the immune system. Critical microbial metabolites include SCFAs, neuroactive molecules, vitamins, epigenetic factors, inflammogens, hormones, and probably hundreds of other still unidentified factors. Influencing organogenesis and maturation of the embryo, and then later the fetus and developing human infant, the early gut microbiome and metabolome may exert effects that are not displayed until later in life, or even in aging. Many of the diseases now posited to be linked with the early gut microbiome occur through the accrual of slowly developing, chronic pathophysiological changes that have their origins in early life. Translating this emerging body of evidence into interventions that interrupt and alter the early gut microbiome is a future strategy.
